# Avian Influenza Virus (H11N9) in Migratory Shorebirds Wintering in the Amazon Region, Brazil

**DOI:** 10.1371/journal.pone.0110141

**Published:** 2014-10-16

**Authors:** Jansen de Araujo, Severino M. de Azevedo Júnior, Nicolas Gaidet, Renata F. Hurtado, David Walker, Luciano M. Thomazelli, Tatiana Ometto, Marina M. M. Seixas, Roberta Rodrigues, Daniele B. Galindo, Adriana C. S. da Silva, Arlinéa M. M. Rodrigues, Leonardo L. Bomfim, Marcelo A. Mota, Maria E. Larrazábal, Joaquim O. Branco, Patricia Serafini, Isaac S. Neto, John Franks, Richard J. Webby, Robert G. Webster, Edison L. Durigon

**Affiliations:** 1 BSL3+ Laboratório de Virologia Clínica e Molecular do Instituto de Ciências Biomédicas (ICB-II), Universidade de São Paulo, São Paulo, Brazil; 2 Departamento de Biologia da Universidade Federal Rural de Pernambuco, Pernambuco, Brazil; 3 CIRAD-ES, UR AGIRS, Montpellier, France; 4 Department of Infectious Diseases, St. Jude Children's Research Hospital, Memphis, Tennessee, United States of America; 5 Agência de Defesa Agropecuária do Estado do Pará- ADEPARA, Pará, Brazil; 6 SFA-PA- Superintendência Federal de Agricultura no estado do Pará. Ministério da Agricultura Pecuária e Abastecimento (MAPA), Distrito Federal, Brasília, Brazil; 7 Biologia Animal da Universidade Federal de Pernambuco, Pernambuco, Brazil; 8 Centro de Ciências Tecnológicas da Terra e do Mar, Universidade do Vale do Itajaí (UNIVALI), Santa Catarina, Brazil; 9 Centro Nacional de Pesquisa e Conservação das Aves Silvestres (CEMAVE), Cabedelo, Paraíba, Brazil; Australian National University, Australia

## Abstract

Aquatic birds are the natural reservoir for avian influenza viruses (AIV). Habitats in Brazil provide stopover and wintering sites for water birds that migrate between North and South America. The current study was conducted to elucidate the possibility of the transport of influenza A viruses by birds that migrate annually between the Northern and Southern Hemispheres. In total, 556 orotracheal/cloacal swab samples were collected for influenza A virus screening using real-time RT-PCR (rRT-PCR). The influenza A virus-positive samples were subjected to viral isolation. Four samples were positive for the influenza A matrix gene by rRT-PCR. From these samples, three viruses were isolated, sequenced and characterized. All positive samples originated from a single bird species, the ruddy turnstone (*Arenaria interpres*), that was caught in the Amazon region at Caeté Bay, Northeast Pará, at Ilha de Canelas. To our knowledge, this is the first isolation of H11N9 in the ruddy turnstone in South America.

## Introduction

Avian influenza viruses (AIV) are globally distributed in wild birds and have been isolated from a wide diversity of avian species [1], [2]. Wild aquatic birds are considered the natural reservoir for AIV [3], and all known hemagglutinin (HA) and neuraminidase (NA) subtypes of AIV have been identified in these species [4]. Charadriiformes, together with Anseriformes, serve as the main reservoir of low pathogenic avian influenza (LPAI) viruses [5]. There are reports showing that LPAI are virus precursors of high pathogenic avian influenza (HPAI) viruses [6]. This relation is of concern because HPAI viruses cause enormous economic losses and have, in rare instances, become major public health issues. For example, deadly outbreaks of Influenza A virus include the H1N1 virus descended from the 1918 ‘Spanish flu’ pandemic, the H2N2 1957 ‘Asian flu’ pandemic strain, the 1968 ‘Hong Kong flu’ strain, the highly pathogenic H5N1 ‘bird flu’ virus of 1997, the 2009 H1N1 pandemic strain [7] and the most recent introduction of H7N9 in China that caused human fatalities [8]. Brazil is considered free from highly pathogenic AIV in poultry, and, being a major global exporter of chicken meat, the emergence of such a disease could have a large economic impact due to trade restrictions [9]. Therefore, information on low-pathogenic viruses circulating among wild birds in Brazil may be useful for improving knowledge for Reference: Most shorebirds are long-distance migratory birds [10]. In the Americas, a large number of migratory shorebirds migrate from North America, where they breed, to South America, where they spend the boreal winter [11]. These birds have the potential to disperse AIV between the Northern and Southern hemispheres as they migrate.

The surveillance of migratory birds for AIV is conducted in several countries of the Pacific and Atlantic flyways in the New World [10]–[14]. However, AIV has been detected in different species, usually at low prevalence [15], with the exception of a high prevalence in ruddy turnstones at Delaware Bay (USA) during the spring migration in May [7]. Delaware Bay represents the only site worldwide where AIV isolations from shorebirds (Scolopacidae) have consistently been reported [5], [7].

**Figure 1:**
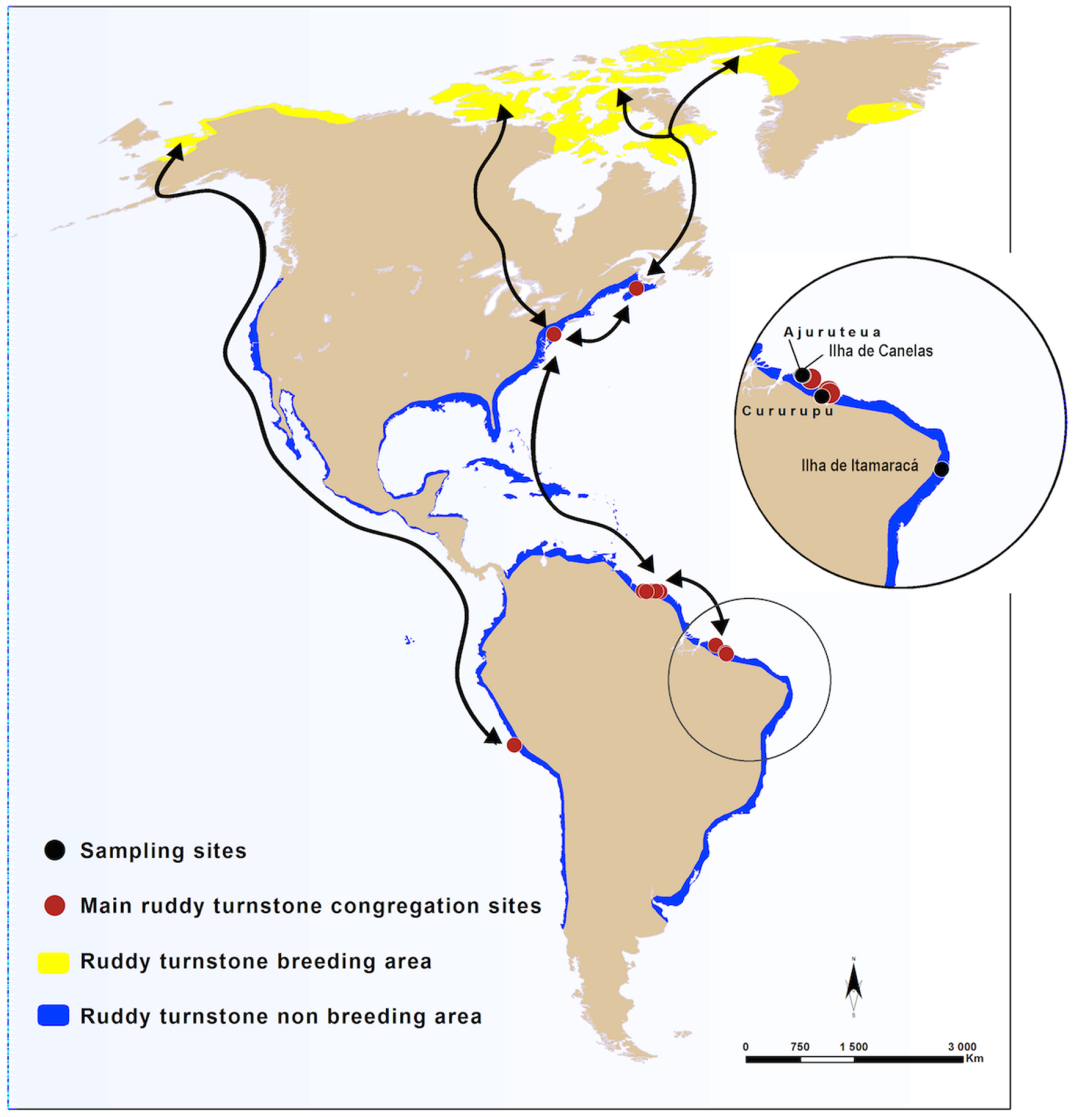
Location of sampling sites along the Brazilian coast in relation to the main migration routes and congregation sites of ruddy turnstones in North and South America. Samples were collected in Jan-May and Aug-Dec from 2006 to 2010. (map by M. Gély ©Cirad, adapted from Wings Over Wetlands UNEP-GEF Flyways Project and [34].

The coast of Brazil offers several important stopover sites and wintering sites for migratory shorebirds, including ruddy turnstones (**Figure 1**). Thus, at least two important questions must be addressed to better understand AIV dispersal: first, whether ruddy turnstones bring AIV from breeding areas in North America to their wintering sites in South America, and second, whether AIV persists in shorebirds in their wintering sites in South America, hence constituting a source of virus introduction to North America during the spring migration.

The seasonal movements of shorebirds are generally associated with migrations to breeding sites or food [16]. Shorebirds migrating from boreal winter arrive in Brazil, usually in September, via routes through the western Amazon, eastern Amazon, Central Plateau Brazil and Atlantic coast and remain in the country until April [17]. During June and July, many species use breeding grounds in the Arctic, and only juveniles, sub-adults or adults that have not completed the molt cycle remain in wintering areas until the next migration season [18]. The largest flocks observed in Brazil are on the northern and southern coasts, and smaller flocks are found on the northeastern and eastern coasts [19]–[23].

Before the emergence of HPAI (H5N1) in Asia (1997), the surveillance of AIV in migratory birds in Brazil was limited. Prior to 2003, only commercial poultry was routinely monitored for AIV due to export regulations from the Brazilian Agriculture Department (MAPA). Wild-bird surveillance was implemented in 2003–2005 within the framework of the Brazilian Ministry of Health [24]. On occasion, the detection of AIV (H3) from Red Knot (*Calidris canutus*), Sanderling (*Calidris alba*), Least Sandpiper (*Calidris minutilla*), White-rumped Sandpiper (*Calidris fuscicollis*), Ruddy Turnstone (*Arenaria interpres*) and Semipalmated Sandpiper (*Calidris pusilla*) sampled on the coast of Brazil was reported [25].

In the present study, shorebirds and sympatric species were selected for influenza A surveillance because of their status as reservoir species. Important sites for migratory birds were selected in accordance with migration patterns and logistical limitations. In the late spring and early autumn of the years 2006 to 2010, birds were sampled and tested for the presence of AIV at several of the main stopover and wintering sites in Brazil to characterize the pattern of dissemination of AIV in shorebirds.

## Material and Methods

### Study sites

Our study was conducted during 2006–2010 with field-sampling operations coordinated by the University of São Paulo (USP), University of Pernambuco (UFRPE), Brazilian Ministry of Agriculture (MAPA), University of Itajaí Valley (UNIVALI) and National Center of Research and Conservation of Wild Birds (CEMAVE). From February 2006 to November 2010, AIV surveillance was conducted at three locations on the Atlantic coast of Brazil: one site in the Amazon region within the state of Pará (Ilha de Canelas) and two sites on the northeastern coast in the states of Maranhão and Pernambuco (villages of Cururupu and Ilha de Itamaracá, respectively) (**Figure 1**).

### Sample collection

We collected orotrachael and cloacal swabs from birds captured by a mist net. Paired orotrachael and cloacal swabs were combined in the same vial with Virus Transport Media (VTM), which consisted of PBS-balanced salt solution supplemented with 0.5% bovine albumin (BSA), antimicrobial agents (200 U/mL penicillin G, 200 U/mL streptomycin, 25 µg/mL fungisone and 6 µg/mL gentamycin) and 10% glycerol. In addition, fresh fecal samples from shorebirds were collected from the ground after identification of the source taxon and placed in vials containing VTM. The samples were stored in dry shippers (liquid nitrogen) and then stored in the laboratory at −80°C until processed.

### Avian influenza virus detection

The samples were analyzed at the Microbiology BSL3^+^ Laboratory at the Biomedical Sciences Institute of the University of São Paulo. RNA was extracted from the orotrachael and cloacal swabs of the birds using the QIAmp RNeasy mini kit (Qiagen Inc., Valencia, CA) according to the manufacturer's instructions. The samples were screened by real-time RT-PCR (rRT-PCR) using an AIV-M TaqMan Reagents Kit (Applied Biosystems, Foster City, CA, USA) [26].

### Virus isolation

All samples that tested positive for the Influenza A virus matrix gene were subsequently submitted to the Department of Infectious Diseases, St. Jude Children's Research Hospital, Memphis, TN, USA for viral isolation using standard methods through inoculation in ten-day-old, embryonated chicken eggs [27]. Each positive isolation was confirmed with an Avian Influenza Virus Type A Test Kit (Flu-DetectH^®^ Synbiotics, San Diego, CA) [28]. Subtyping was performed using hemagglutination and neuraminidase inhibition tests and a panel of polyclonal reference sera [29]–[31]. In addition, sequencing of the HA and NA genes of the isolates was performed.

### RNA extraction and sequencing

Viral RNA was extracted from 400 µL of allantoic fluid with an RNeasy Mini Kit (Qiagen, Hilden, Germany) according to the manufacturer's instructions. Extracted RNA was eluted in 80 µL of RNase-free water. The cDNA was transcribed using a High-Capacity cDNA Archive Kit (Applied Biosystems, Foster City, CA, USA) according to the manufacturer's directions. The full-length HA (1778 bp) and NA (1413 bp) genomic segments were sequenced using specific primers (**Table 1** **and** **Table 2**) on an ABI PRISM 3100 Genetic Analyzer (Applied Biosystems/Life Technologies) and the Big Dye Terminator v3.1 Cycle Sequencing Kit (Life Technologies) at the Hartwell Center for Bioinformatics and Biotechnology at St. Jude Children's Research Hospital (Memphis, TN, USA).

**Table 1:** Primers used to sequence the complete HA gene.

Sequence	Oligonucleotide Position (nt)
5′ GGA GTT CAC CAT CCT GCA ACT CT 3′	H11-580
5′ TTC CTT TAG AGT TGC AGG ATG GTG 3′	H11-600R
5′ AAC CAC CTT TCA TGA AAC CAG CTA TTG C 3′	H11-1080R
5′ ACA GCT TCT CGT TTT ACT TGA AAA TGA 3′	H11-1330
5′ CAT TTT CAA GTA AAA CGA GAA GCT GTG CA 3′	H11-1350R

**Table 2:** Primers used to sequence the complete NA gene.

Sequence	Oligonucleotide Position (nt)
5′ TAT TCG TCT CAG GGA GCA AAA GCA GGG TC 3′	‘Bm-N9-1
5′ATA TCG TCT CGT ATT AGT AGA AAC AAG GGT CTT 3′	‘Bm-N9-1467R
5′ ATA AAT TCA TGG CAC ATA TAT GG 3′	N9×300
5′ GTC AAG TAC TAG TTG CCA TGA TG 3′	N9×560
5′ CAT CAT GGC AAC TAG TAC TTG AC 3′	N9×580R
5′ GGA TAA TTG GCA GGG CTC AAA TAG 3′	N9×900
5′ GAT CTT GAA GCT GTG CTT ATT GT 3′	N9×1137R

### Genetic characterization and phylogenetic tree of the HA and NA genes

Nucleotide-level searches were performed for each genome segment of each virus isolate using the Basic Local Alignment Search Tool (BLAST) to identify the most closely related sequences available in public databases [32]. The sequences were initially aligned using the Bioedit Sequence Alignment Editor (v.7.1.5.0) (Ibis Biosciences Carlsbad, CA), and the nucleotide percentage similarities were calculated using the MegAlign program of the DNASTAR package (DNASTAR, Inc.).

To elucidate the relationships between the viruses isolated in this study, a phylogenetic analysis of the HA and NA genes, including all available H11 and N9 subtype influenza gene sequences between 2007–2009 in North America and other regions was performed. Phylogenetic analyses of the HA and NA genes were performed for specific-subtype group characterization using only the open reading frames (ORFs) of each segment, and the trees were generated by PAUP 4.0b10 using the maximum likelihood algorithm with the parameters indicated by the GTR Modeltest. Levels of bootstrap are shown for major clades [33] The sequences of the segments from the three isolates of influenza A virus were deposited with the following GenBank accession numbers for HA: KF824501 (A08), KF824502 (A17) and KF824503 (A51) and NA: KF824504 (A08), KF824505 (A17) and KF824506 (A51).

### Ethics Statement

All procedures involving wild birds were approved by the Brazilian Society of Laboratory Animal Science (SBCAL) of the University of Sao Paulo (Permit Number: 105, page 74, book 2) and licensed by the Ministério do Meio Ambiente-MMA at the Instituto Chico Mendes de Conservação da Biodiversidade (ICMBio/SISBIO), Number 25895-1.

## Results

A total of 556 birds from 23 different species, representing 14 different families of the order Charadriiformes, were sampled (**Table 3**). Most of these species were migratory. AIV was only detected in ruddy turnstones (n = 4/68), all in Ilha de Canelas, Amazon region (Pará State) at the beginning of the wintering period in November 2008, using rRT-PCR amplification of the influenza A matrix gene. The proportion of ruddy turnstone that was infected with AIV was 5.8%. Of the four influenza A-positive samples, virus was isolated from three. BLAST searches of the surface glycoprotein genes of the three isolates from ruddy turnstones confirmed 94 to 99% sequence similarity to HA of the H11 subtypes, and all three isolates were 99% similar to Influenza A virus (A/ruddy turnstone/New Jersey/AI09-194/2009 (H11N8); ID: CY145907-1). The isolates were also 94 to 99% similar to NA of subtype N9 and 99% similar to Influenza A virus (A/shorebird/Delaware Bay/351/2009(H1N9); ID: CY137916.1) (**Table 4** **and** **Table 5**).

**Table 3:** Surveillance of influenza A viruses in wild birds in Brazil by real-time RT-PCR.

Site	Date	Scientific name	Sample*	No. pos./No. birds
Ilha de Canelas, PA	Nov. 06	*Actitis macularius*	C & O	0/10
		*Calidris canutus*	C & O	0/1
		*Calidris pusilla*	C & O	0/3
		*Calidris minutilla*	C & O	0/1
		*Sterna hirundo*	C & O	0/4
		*Larus* sp.	C & O	0/1
Ajuruteua, PA	Nov. 06	*Actitis macularius*	C & O	0/5
		*Calidris pusilla*	C & O	0/2
		*Charadrius semipalmatus*	C & O	0/1
Ilha de Itamaracá, PE	Apr. 07	*Arenaria interpres*	C & O	0/7
		*Calidris alba*	C & O	0/8
		*Calidris pusilla*	C & O	0/5
		*Charadrius semipalmatus*	C & O	0/3
		*Limnodromus griseus*	C & O	0/1
		*Sterna hirundo*	C & O	0/1
Ilha de Itamaracá, PE	Aug. 08	*Charadrius semipalmatus/C. alba/A. interpres*	F	0/100
		*Sterna hirundo/Thalasseus sandvicensis*	F	0/64
Ilha de Itamaracá, PE	Oct. 08	*Arenaria interpres*	C & O	0/1
		*Calidris pusilla*	C & O	0/1
		*Calidris pusilla*	C & O	0/5
		*C. pusilla/A. interpres/C. alba/Pluvialis squatarola*	F	0/18
Ilha de Canela, PA	Nov. 08	*Actitis macularius*	C & O	0/31
		***Arenaria interpres***	**C & O**	**4/22**
		*Calidris alba*	C & O	0/2
		*Limnodromus griseus*	C & O	0/9
		*Tringa melanoleuca*	C & O	0/1
		*Charadrius semipalmatus*	C & O	0/1
		*Gelochelidon nilotica*	C & O	0/1
Cururupu, MA	May. 10	*Actitis macularius*	C & O	0/27
		*Arenaria interpres*	C & O	0/12
		*Calidris pusilla*	C & O	0/98
		*Calidris canutus*	C & O	0/7
		*Limnodromus griseus*	C & O	0/1
		*Vanellus chilensis*	C & O	0/10
		*Charadrius collaris*	C & O	0/6
		*Charadrius semipalmatus*	C & O	0/6
		*Haematopus palliatus*	C & O	0/6
		*Himantopus mexicanus*	C & O	0/25
		*Chroicocephalus maculipennis*	C & O	0/1
		*Gelochelidon nilotica*	C & O	0/4
		*Stercorarius skua*	C & O	0/2
		*Jacana*	C & O	0/2
Ilha de Itamaracá, PE	Oct. 10	*Arenaria interpres*	C & O	0/25
		*Calidris pusilla*	C & O	0/11
		*Calidris alba*	C & O	0/1
		*Sterna hirundo*	C & O	0/3
Total		*21 species*		4/556

*Sample type: C =  Cloacal, O =  Oral and F =  Feces.

**Table 4:** The closest sequences to HA of influenza A virus available in public databases based on percent nucleotide similarity.

GenBank ID	Sequence name	A08/2008 (H11) [208614]	A17/2008 (H11) [208615]	A51/2008 (H11) [208617]
CY145907.1	A/ruddy turnstone/New Jersey/AI09-194/2009(H11N8)	99%	99%	99%
CY145437.1	A/ruddy turnstone/New Jersey/Sg-00557/2008(H11N5)	99%	99%	99%
CY145711.1	A/ruddy turnstone/New Jersey/Sg-00564/2008(H11N9)	99%	99%	99%
CY127871.1	A/shorebird/Delaware Bay/257/2009(H11N8)	99%	99%	99%
CY127887.1	A/shorebird/Delaware Bay/549/2009(H11N1)	99%	99%	99%
CY145719.1	A/ruddy turnstone/New Jersey/Sg-00567/2008(H11N2)	99%	99%	99%
CY145955.1	A/herring gull/New Jersey/AI09-335/2009(H11N1)	99%	99%	99%
CY138137.1	A/lesser black-backed gull/Iceland/145/2010(H11N2)	98%	98%	98%
CY039532.1	A/green winged teal/California/AKS1305/2008(H11N9)	97%	97%	97%
KF542875.1	A/Anas acuta/New Mexico/A00629381/2008(H11N9)	96%	96%	96%
CY096624.1	A/blue-winged teal/Guatemala/CIP049-10/2009(H11N2)	95%	95%	95%
HM193587.1	A/mallard/Alaska/44430-056/2008(H11N9)	95%	95%	95%
CY127638.1	A/mallard/Alberta/193/2000(H11N9)	94%	94%	94%

**Table 5:** The closest sequences to NA of influenza A virus available in public databases based on percent nucleotide similarity.

GenBank ID	Sequence name	A08/2008 (N9) [208614]	A17/2008 (N9) [208615]	A51/2008 (N9) [208617]
CY137916.1	(A/shorebird/Delaware Bay/351/2009(H1N9)	99%	99%	99%
CY146281.1	A/ruddy turnstone/New Jersey/AI09-1082/2009(H1N9)	99%	99%	99%
CY145689.1	A/ruddy turnstone/New Jersey/Sg-00561/2008(H11N9)	99%	99%	99%
CY146289.1	A/ruddy turnstone/New Jersey/AI09-1144/2009(H11N9)	99%	99%	99%
CY038237.1	A/ruddy turnstone/New Jersey/Sg-00561/2008(H11N9)	99%	99%	99%
CY125351.1	A/ring-necked duck/New Brunswick/03449/2009(H11N9)	98%	98%	98%
CY004368.1	A/mallard/Alberta/245/2003(H11N9)	97%	97%	97%
CY144334.1	A/red knot/Delaware/650666/2002(H11N9)	96%	96%	96%
JF323789.1	A/Steller's eider/Alaska/44422-253/2008(H11N9)	94%	94%	94%

**Figure 2:**
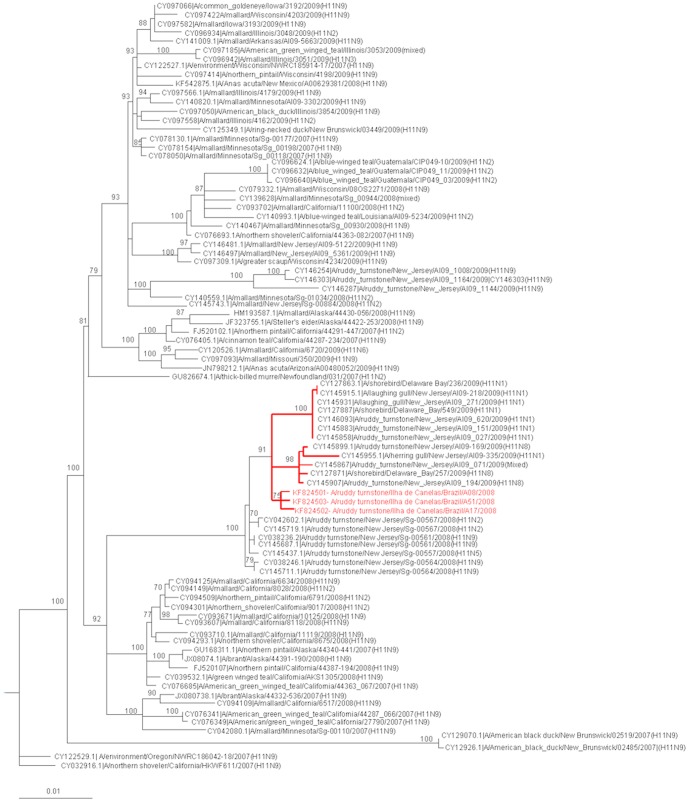
Phylogenetic analysis of the hemagglutinin gene of the influenza A virus. Trees were generated by PAUP 4.0b10 using the maximum likelihood algorithm with the parameters indicated by the GTR Modeltest (bootstrap values are shown for the branch points). The scale bar is shown on the bottom left. In this study, sequences that were available in the public database were used to construct the tree, and the accession numbers along with their branch data are shown.

**Figure 3:**
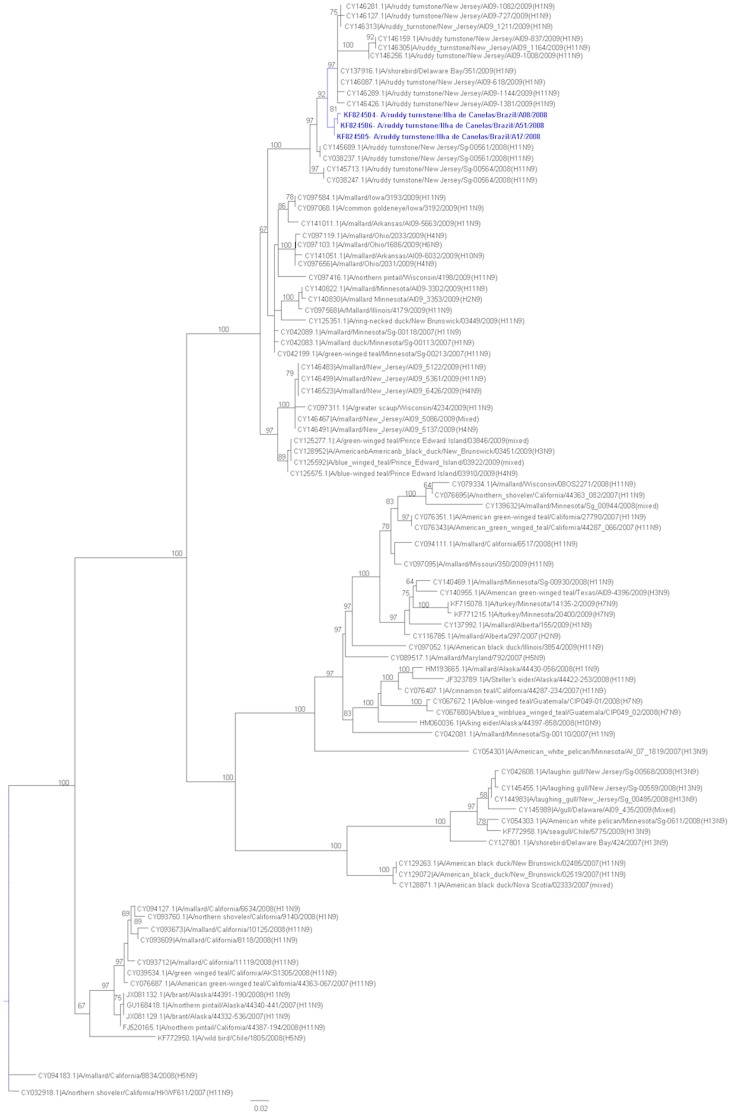
Phylogenetic analysis of neuraminidase of the influenza A virus. Trees were generated by PAUP 4.0b10 using the maximum likelihood algorithm with the parameters indicated by the GTR Modeltest (bootstrap values are shown for the branch points). The scale bar is shown on the bottom left. In this study, sequences that were available in public databases were used to construct the tree, and the accession numbers along with their branch data are shown.

The high similarity between the isolates and the formation of a clade indicates that these isolates are closely related. The HA and NA genes segments were most closely related to North American avian lineage viruses from New Jersey and Delaware Bay from 2009 (99% identity). The phylogenetic tree shows that these isolates are grouped in a different cluster from others H11 and N9 subtypes viruses and supported with high bootstrap values (**Figure 2** **and** **Figure 3**). In addition, we analyzed the isolates with sequences available from other continents (see **Figure S1 and Figure S2**).

## Discussion

Ilha de Canelas is an important stopover for migratory shorebirds in South America [34] and the only site in our study where AIV was detected. During our surveillance sampling effort, we detected AIV only in ruddy turnstones, which was consistent with other studies that found ruddy turnstones to be frequently infected with AIV [5], [7]. Reports indicate that AIV may occur at higher rates in ruddy turnstones than in other Scolopacidae [15]. H11 in combination with various NA subtypes has been reported throughout 2000–2010 in Asia [35], [36], Europe [37]–[39], the USA and Canada [40] as well as Central America [14]. It is therefore not surprising to find H11 in Brazil.

Previous outbreaks of highly pathogenic influenza in the Americas have originated from the introduction of low-pathogenic H5 or H7 precursors making their way into poultry populations with subsequent evolution to a highly pathogenic form [40]. Although these two subtypes of greatest concern have not been detected in Brazil, the low number of subtypes that have been isolated in comparison with other countries raises the question as to whether the current surveillance strategy is adequately determining the full range of subtypes in the region. Thus, exploration of other possible sampling sites and species is warranted.

Our results suggest that it is possible for ruddy turnstones to carry AIV from the northern hemisphere to their wintering sites in South America. The virus was only detected during the arrival of the birds in Brazil, and we did not detect AIV in other shorebird species that occur in sympatry with the ruddy turnstones in Brazil. This raises the question as to whether there are inter-species transmission barriers among shorebird species. H11 was not a predominant HA subtype detected in shorebirds at Delaware Bay [15], [41]. However, H11N9 AIVs have been detected (2002–2004, 2007–2009) in shorebirds at Delaware Bay in long-term monitoring studies. The existence of shared AIV subtypes in shorebirds between the South and North American staging sites suggests that virus exchange may occur between the two continents.

*Arenaria interpres* is a long-distance migratory bird with circumpolar distribution. This species breeds in northeastern Alaska and the Canadian Arctic and then moves along the Pacific and Atlantic coasts toward Central and South America [11]. Delaware Bay represents a globally important site where AIV isolations from shorebirds (Scolopacidae) have consistently been reported [7], [32]. Previous work showed that ruddy turnstone (*Arenaria interpres*) represented 40% of all AIV isolates, even though only 11.2% of the sampled birds were of the Charadriiformes order [15], [42]. The combination of HA and NA that was most frequently isolated from shorebirds included H3N8 (7.1%) and H11N9 (7.6%) [43].

Our work was performed over five years during two distinct time periods: the time of arrival of birds from the Northern Hemisphere in November and the time of return in April [44]. Our results confirm the presence of low-pathogenic influenza A virus in wild birds during the arrival period in Brazil. The phylogenetic analysis of the HA and NA viral sequences revealed that the AIV isolates are of the H11N9 group and exhibit high similarity (99%) to viruses of the North American lineage found in shorebirds. One limitation of this study is not including the full virus genomes that would allow for more in depth molecular characterization for a better understanding of the eco-epidemiology and transcontinental transport of these viruses.

In Brazil, a small number of groups have worked with AIV in wild birds. Extensive data on the patterns of AI virus infection in wild birds only exist for Europe and North America [3], [37], where the majority of surveillance has traditionally been focused, and similar information for many parts of the Southern Hemisphere is lacking [5], [45]. The patterns of AIV circulation that are observed in Europe and North America cannot be directly transposed to the Southern Hemisphere, particularly to tropical regions, where differences in host ecology, climate and seasonality may produce different dynamics of infection [45].

The presence of AIV in wild birds in South America has been confirmed, and the detection or isolation of LPAI viruses in the following countries has been shown: subtypes H13N9, H1N1 and H6N2 [46]–[48] were isolated in Argentina; H7N3 was isolated in Bolivia and Chile [40], [49]; H3 was isolated in Brazil [25], [50]; H5N2 was isolated in Colombia [51]; and H3N8, H4N5, H10N9 and H13N2 were isolated in Peru [13], [52], [53].

## Conclusion

An effective surveillance program for Influenza A viruses in migratory bird species may alert public health authorities of potential human and domestic animal pathogens and facilitate the implementation of response measures in Brazil.

The results of our surveillance of migratory birds demonstrated high similarity in the HA and NA genes to viruses of the North American lineage found in shorebirds.

## Supporting Information

Figure S1 **Phylogenetic analysis of the hemagglutinin gene of the influenza A virus with sequences available from other continents.** The accession numbers along with their branch data are shown.
(TIFF)

Figure S2 **Phylogenetic analysis of neuraminidase of the influenza A virus with sequences available from other continents.** The scale bar is shown on the bottom left. The accession numbers along with their branch data are shown.
(TIFF)

## Biographical Sketch

Dr. Jansen de Araujo has a Ph.D. in Microbiology and is the coordinator of the field team in the Virology Laboratory at the Biomedical Science Institute. His research interests include ecoepidemiological studies of wildlife and emerging infectious diseases with zoonotic potential in Brazil.

## References

[1] Olsen B, Munster VJ, Wallensten A, Waldenström J, Osterhaus ADME, et al (2006) Global patterns of influenza a virus in wild birds. Science 312: 384–388. 16627734 10.1126/science.1122438

[2] Brown JD, Luttrell MP, Berghaus RD, Kistler W, Keeler SP, et al (2010) Prevalence of antibodies to type a influenza virus in wild avian species using two serologic assays. J Wildl Dis 46: 896–911. 20688695 10.7589/0090-3558-46.3.896 PMC11345711

[3] Stallknecht DE (2007) Impediments to wildlife disease surveillance, research, and diagnostics. Curr Top Microbiol Immunol 315: 445–461. 17848074 10.1007/978-3-540-70962-6_17

[4] Beare AS, Webster RG (1991) Replication of avian influenza viruses in humans. Arch Virol 119: 37–42. 1863223 10.1007/BF01314321

[5] Gaidet N, Caron A, Cappelle J, Cumming GS, Balança G, et al (2012) Understanding the ecological drivers of avian influenza virus infection in wildfowl: a continental-scale study across Africa. Proc Biol Sci 279: 1131–1141. 21920984 10.1098/rspb.2011.1417 PMC3267134

[6] Pasick J, Handel K, Robinson J, Copps J, Ridd D, et al (2005) Intersegmental recombination between the haemagglutinin and matrix genes was responsible for the emergence of a highly pathogenic H7N3 avian influenza virus in British Columbia. J Gen Virol 86: 727–731. 15722533 10.1099/vir.0.80478-0

[7] Krauss S, Stallknecht DE, Negovetich NJ, Niles LJ, Webby RJ, et al (2010) Coincident ruddy turnstone migration and horseshoe crab spawning creates an ecological “hot spot” for influenza viruses. Proc Biol Sci 277: 3373–3379. 20630885 10.1098/rspb.2010.1090 PMC2982236

[8] Chen E, Chen Y, Fu L, Chen Z, Gong Z, et al.. (2013) Human infection with avian influenza A(H7N9) virus re-emerges in China in winter 2013. Euro Surveill 18: pii: 20616. 10.2807/1560-7917.es2013.18.43.20616 24176616

[9] Thomazelli L, Araujo J de, Ferreira C de S, Hurtado R, Oliveira D, et al (2012) Molecular surveillance of the Newcastle disease virus in domestic and wild birds on the North Eastern Coast and Amazon biome of Brazil. Rev Bras Ciência Avícola 14: 01–07.

[10] Pearce JM, Ramey AM, Ip HS, Gill RE (2010) Limited evidence of trans-hemispheric movement of avian influenza viruses among contemporary North American shorebird isolates. Virus Res 148: 44–50. 19995585 10.1016/j.virusres.2009.12.002

[11] Rodrigues RC, Azevedo-Júnior SM de, Larrazábal MEL de, Araujo HFP de (2009) Temporal variations of body mass and plumage in Arenaria interpres (Aves: Scolopacidae) along the Brazilian coast. Zool (Curitiba, Impresso) 26: 386–390.

[12] D'Amico VL, Bertellotti M, Baker AJ, Diaz L a (2007) Exposure of red knots (Calidris canutus rufa) to select avian pathogens; Patagonia, Argentina. J Wildl Dis 43: 794–797. 17984283 10.7589/0090-3558-43.4.794

[13] Ghersi BM, Blazes DL, Icochea E, Gonzalez RI, Kochel T, et al (2009) Avian influenza in wild birds, central coast of Peru. Emerg Infect Dis 15: 935–938. 19523296 10.3201/eid1506.080981 PMC2727326

[14] González-Reiche AS, Morales-Betoulle ME, Alvarez D, Betoulle J-L, Müller ML, et al (2012) Influenza a viruses from wild birds in Guatemala belong to the North American lineage. PLoS One 7: e32873. 22427902 10.1371/journal.pone.0032873 PMC3302778

[15] Hanson BA, Luttrell MP, Goekjian VH, Niles L, Swayne DE, et al (2008) Is the occurrence of avian influenza virus in Charadriiformes species and location dependent? J Wildl Dis 44: 351–361. 18436667 10.7589/0090-3558-44.2.351

[16] Sick H (1997) Espécies visitantes, migrações. Ornitologia Brasileira: Uma Introdução. Brasília: Universidade de Brasília. pp. 129–131.

[17] Antas PTZ (1983) Migration of Neartic shorebirds (Charadriidae and Scolopacidae) in Brazil – flyways and their different seazonal use. Wader Study Gr Bull 39: 51–56.

[18] Azevedo Júnior SM, Dias MM, Larrazábal ME de, Telino Júnior WR, Lyra-Neves RM, et al (2001) Recapturas e recuperações de aves migratórias no litoral de Pernambuco, Brasil. Ararajuba 9: 33–42.

[19] Morrison RIG, Ross RK (1989) Atlas of nearctic Shorebirds on the Coast of South America. Can Wildl Serv 2: 128.

[20] Lara-Resende S de M (1982) Recuperação de anilhas estrangeiras no Brasil. Rev Bras Zool 1: 231–237.

[21] Larrazábal M, Azevedo-Júnior SM, Pena O (2002) Monitoramento de aves límicolas na Salina Diamante Branco, Galinhos, Rio Grande do Norte, Brasil. Rev Bras Zool 19: 1081–1089.

[22] Telino-Júnior WR, Azevedo-Júnior SM DE, Lyra-Neves RM (2003) Censo de aves migratórias 2 (Charadriidae, Scolopacidae e Laridae) na Coroa do Avião, Igarassú, Pernambuco, Brasil. Rev Bras Zool 21: 451–456.

[23] Lyra-Neves RM DE, Azevedo Júnior SM de, Telino-Júnior WR (2004) Monitoramento do maçarico-branco, Calidris alba (Pallas) (Aves, Scolopacidae), através de recuperações de anilhas coloridas, na Coroa do Avião, Igarassu, Pernambuco, Brasil. Rev Bras Zool 21: 319–324.

[24] Secretaria de Vigilância em Saúde (2005) Plano de preparação do Brasil para o enfrentamento de uma pandemia de influenza: p. 223.

[25] Araújo FAA, Vianna R da ST, Andrade Filho GV de, Melhado DL, Todeschini B, et al (2004) Segundo inquérito sorológico em aves migratórias e residentes do Parque Nacional da Lagoa do Peixe/RS para detecção do vírus da Febre do Nilo Ocidental e outros vírus. Bol Eletronico Epidemiol 5: 1–8.

[26] Curd E, Pollinger J, Toffelmier E, Smith T (2011) Rapid influenza A detection and quantitation in birds using a one-step real-time reverse transcriptase PCR and High Resolution Melting. J Virol Methods 176: 125–130. 21663763 10.1016/j.jviromet.2011.05.033

[27] Shortridge KF, Butterfield WK, Webster RG, Campbell CH (1977) Isolation and characterization of influenza A viruses from avian species in Hong Kong. Bull World Health Organ 55: 15–20. 302152 PMC2366618

[28] Das A, Spackman E, Thomas C, Swayne DE, Suarez DL (2008) Detection of H5N1 high-pathogenicity avian influenza virus in meat and tracheal samples from experimentally infected chickens. Avian Dis 52: 40–48. 18459294 10.1637/8093-082107-Reg

[29] Aymard-Henry M, Coleman MT, Dowdle WR, Laver WG, Schild GC, et al (1973) Influenzavirus neuraminidase and neuraminidase-inhibition test procedures. Bull World Health Organ 48: 199–202. 4541685 PMC2481006

[30] Palmer DF, Dowdle WR, Coleman MT, Schild GC (1975) Advanced laboratory techniques for influenza diagnosis. Immunology series no 6. Atlanta: Center for Disease Control, US Department of Health Education and Welfare.

[31] Webster RG, Isachenko VA, Carter M (1974) A new avian influenza virus from feral birds in the USSR: recombination in nature? Bull World Health Organ 51: 325–332. 4549487 PMC2366305

[32] Zhang Z, Schwartz S, Wagner L, Miller W (2000) A greedy algorithm for aligning DNA sequences. J Comput Biol 7: 203–214. 10890397 10.1089/10665270050081478

[33] Swofford DL (2002) PAUP*. phylogenetic analysis using parsimony (*and other methods). Version 4. Sunderland, MA: Sinauer Associates.

[34] Blanco DE, Bernabé LL, Baigún RJ (2007) Mapping waterbird distribution and migration in South America. Wetlands International, South America Programme, Buenos Aires, Argentina. Wetl Int: 1–24.

[35] Okamatsu M, Nishi T, Nomura N, Yamamoto N, Sakoda Y, et al (2013) The genetic and antigenic diversity of avian influenza viruses isolated from domestic ducks, muscovy ducks, and chickens in northern and southern Vietnam, 2010–2012. Virus Genes 47: 317–329. 23861018 10.1007/s11262-013-0954-7

[36] Karamendin K, Kydyrmanov A, Zhumatov K, Asanova S, Ishmukhametova N, et al (2011) Phylogenetic analysis of avian influenza viruses of H11 subtype isolated in Kazakhstan. Virus Genes 43: 46–54. 21461588 10.1007/s11262-011-0603-y

[37] Munster VJ, Baas C, Lexmond P, Waldenström J, Wallensten A, et al (2007) Spatial, temporal, and species variation in prevalence of influenza A viruses in wild migratory birds. PLoS Pathog 3: e61. 17500589 10.1371/journal.ppat.0030061 PMC1876497

[38] Slavec B, Krapez U, Racnik AJ, Hari A, Wernig JM, et al (2012) Surveillance of influenza A viruses in wild birds in Slovenia from 2006 to 2010. Avian Dis 56: 999–1005. 23402126 10.1637/10175-041012-ResNote.1

[39] Hoye BJ, Munster VJ, Nishiura H, Klaassen M, Fouchier R a M (2010) Surveillance of wild birds for avian influenza virus. Emerg Infect Dis 16: 1827–1834. 21122209 10.3201/eid1612.100589 PMC3294547

[40] Senne DA (2007) Avian influenza in North and South America, 2002–2005. Avian Dis 51: 167–173. 17494549 10.1637/7621-042606R1.1

[41] Stallknecht DE, Luttrell MP, Poulson R, Goekjian V, Niles L, et al (2012) Detection of avian influenza viruses from shorebirds: evaluation of surveillance and testing approaches. J Wildl Dis 48: 382–393. 22493113 10.7589/0090-3558-48.2.382 PMC3584701

[42] Kawaoka Y, Chambers TM, Sladen WL, Webster RG (1988) Is the gene pool of influenza viruses in shorebirds and gulls different from that in wild ducks? Virology 163: 247–250. 3348002 10.1016/0042-6822(88)90260-7

[43] Krauss S, Walker D, Pryor SP, Niles L, Chenghong L, et al (2004) Influenza A viruses of migrating wild aquatic birds in North America. Vector Borne Zoonotic Dis 4: 177–189. 15631061 10.1089/vbz.2004.4.177

[44] Kober K (2004) Foraging ecology and habitat use of wading birds and shorebirds in the mangrove ecosystem of the Caeté Bay, Northeast Pará, Brazil Bremen University.

[45] Gaidet N, Ould El Mamy AB, Cappelle J, Caron A, Cumming GS, et al (2012) Investigating avian influenza infection hotspots in old-world shorebirds. PLoS One 7: e46049. 23029383 10.1371/journal.pone.0046049 PMC3460932

[46] Pereda AJ, Uhart M, Perez AA, Zaccagnini ME, La Sala L, et al (2008) Avian influenza virus isolated in wild waterfowl in Argentina: evidence of a potentially unique phylogenetic lineage in South America. Virology 378: 363–370. 18632129 10.1016/j.virol.2008.06.010 PMC2570041

[47] Alvarez P, Mattiello R, Rivailler P, Pereda A, Davis CT, et al (2010) First isolation of an H1N1 avian influenza virus from wild terrestrial non-migratory birds in Argentina. Virology 396: 76–84. 19896684 10.1016/j.virol.2009.10.009

[48] Rimondi A, Xu K, Craig MI, Shao H, Ferreyra H, et al (2011) Phylogenetic analysis of H6 influenza viruses isolated from rosy-billed pochards (Netta peposaca) in Argentina reveals the presence of different HA gene clusters. J Virol 85: 13354–13362. 21976652 10.1128/JVI.05946-11 PMC3233172

[49] Spackman E, McCracken KG, Winker K, Swayne DE (2007) An avian influenza virus from waterfowl in South America contains genes from North American avian and equine lineages. Avian Dis 51: 273–274. 17494565 10.1637/7529-032106R.1

[50] Kawamoto A, Mancini D (2005) Investigation of influenza in migrating birds, the primordial reservoir and transmitters of influenza in Brazil. Brazilian J … 1997: 88–93.

[51] Karlsson EA, Ciuoderis K, Freiden PJ, Seufzer B, Jones JC, et al (2013) Prevalence and characterization of influenza viruses in diverse species in Los Llanos, Colombia. Emerg Microbes Infect 2: e20. 26038461 10.1038/emi.2013.20 PMC3636595

[52] Ghersi BM, Sovero MM, Icochea E, Gonzalez RI, Blazes DL, et al (2011) Isolation of low-pathogenic H7N3 avian influenza from wild birds in Peru. J Wildl Dis 47: 792–795. 21719856 10.7589/0090-3558-47.3.792

[53] Williams RAJ, Segovia-Hinostroza K, Ghersi BM, Gonzaga V, Peterson AT, et al (2012) Avian Influenza infections in nonmigrant land birds in Andean Peru. J Wildl Dis 48: 910–917. 23060492 10.7589/2011-02-052

