# Supplementary figures and images for: Avian Influenza Virus (H11N9) in Migratory Shorebirds Wintering in the Amazon Region, Brazil
Source: PLoS One. 2014 Oct 16;9(10):e110141. doi: 10.1371/journal.pone.0110141 (PMC4199675; doi:10.1371/journal.pone.0110141)

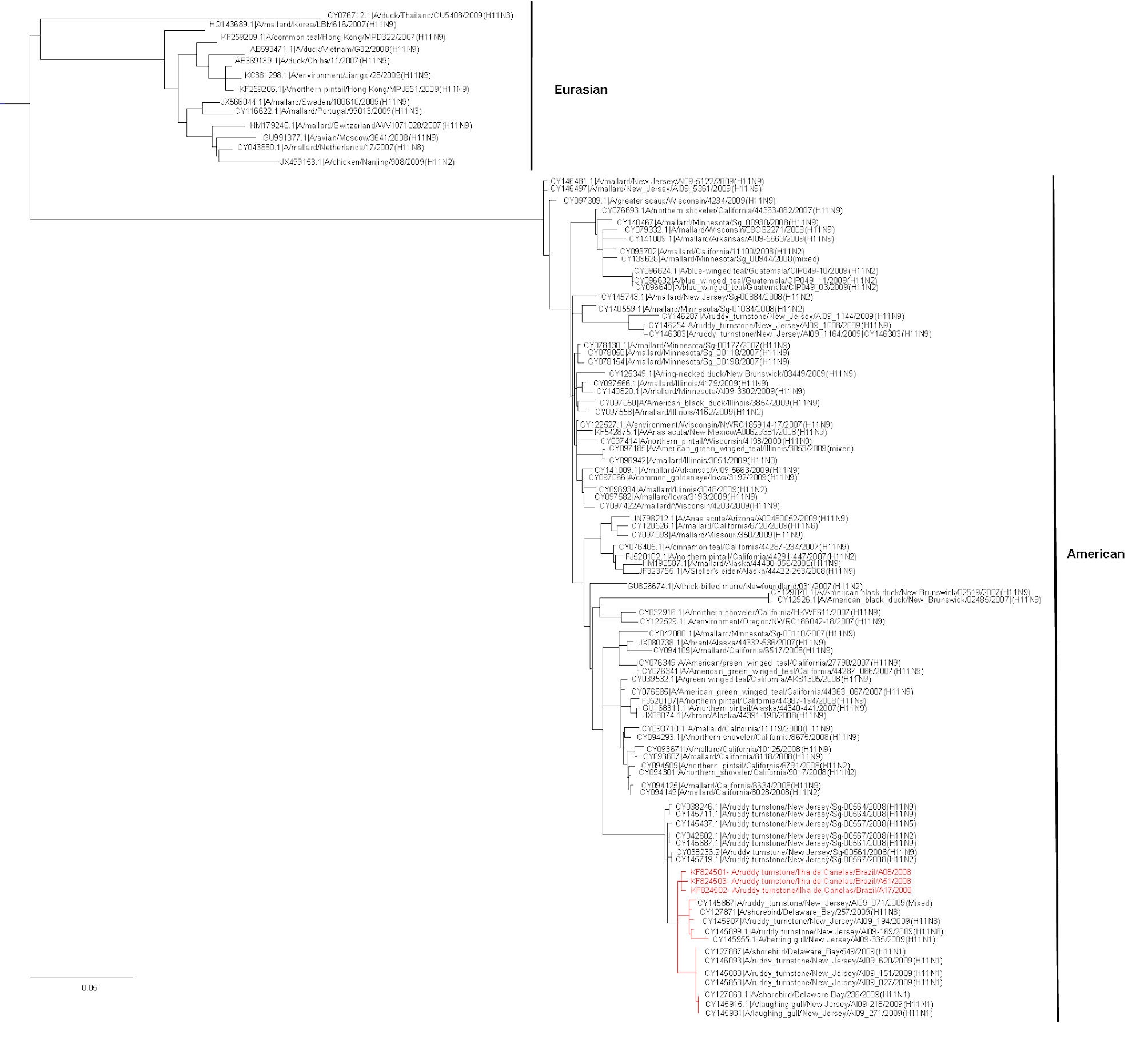

Supplement: Figure S1 — Phylogenetic analysis of the hemagglutinin gene of the influenza A virus with sequences available from other continents. The accession numbers along with their branch data are shown. (TIFF) [file pone.0110141.s001.tiff]

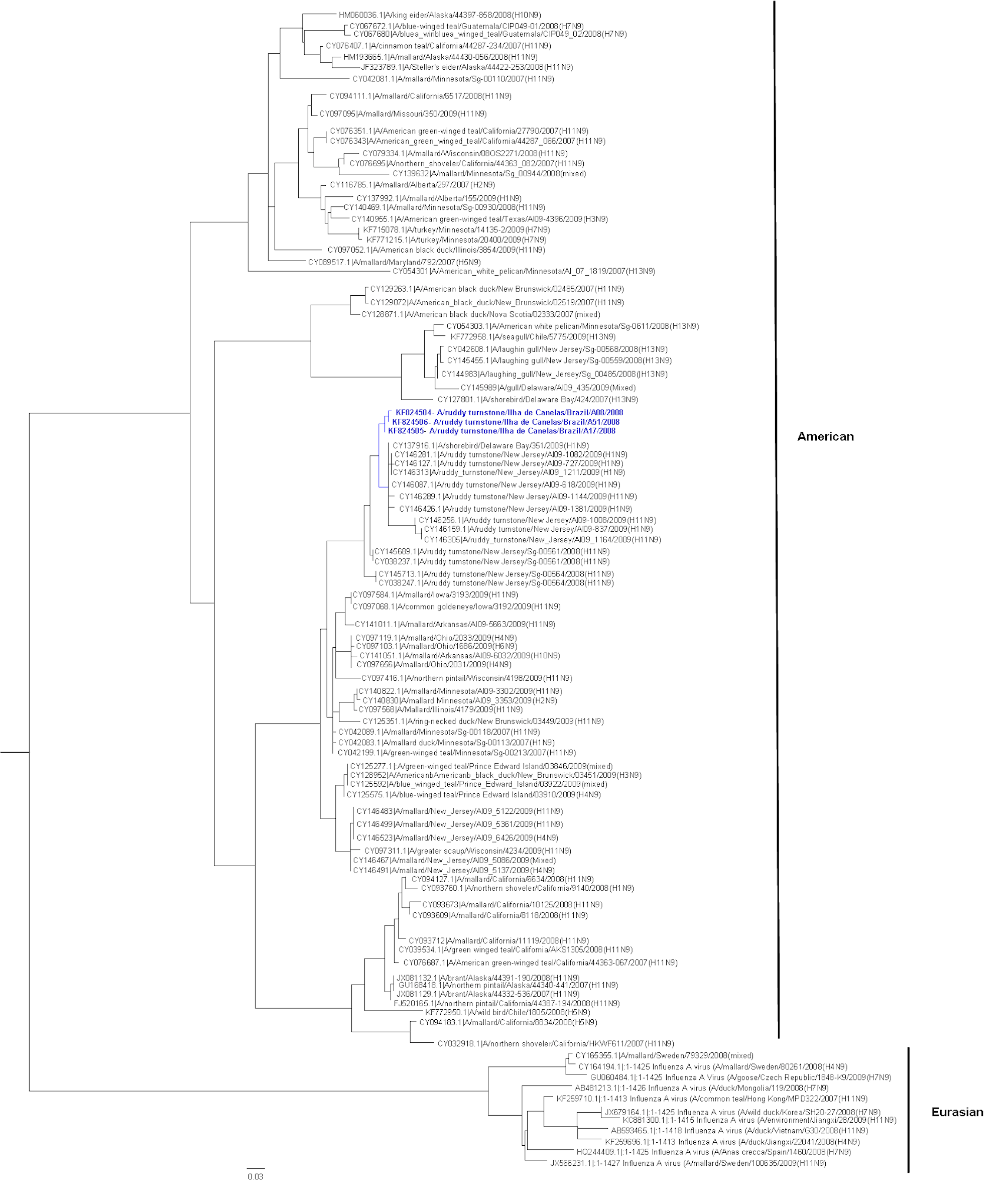

Supplement: Figure S2 — Phylogenetic analysis of neuraminidase of the influenza A virus with sequences available from other continents. The scale bar is shown on the bottom left. The accession numbers along with their branch data are shown. (TIFF) [file pone.0110141.s002.tiff]
